# Application of Computational Intelligence to Improve Education in Smart Cities

**DOI:** 10.3390/s18010267

**Published:** 2018-01-18

**Authors:** Everton Gomede, Fernando Henrique Gaffo, Gabriel Ulian Briganó, Rodolfo Miranda de Barros, Leonardo de Souza Mendes

**Affiliations:** 1 Electrical Engineering and Computer College, State University of Campinas, Av. Albert Einsten, 400, Cidade Universitária Zeferino Vaz, Distrito Barão Geraldo, Campinas 13083-852 SP, Brazil; f163856@dac.unicamp.br (F.H.G.); g151581@dac.unicamp.br (G.U.B.); lmendes@decom.fee.unicamp.br (L.d.S.M.); 2 Computer Science Department, State University of Londrina, Rod. Celso Garcia Cid, Km 380, s/n, Campus Universitário, Londrina 86057-970 PR, Brazil; rodolfo@uel.br

**Keywords:** computational intelligence, smart education, smart city, adaptive learning, creative school, self-learning

## Abstract

According to UNESCO, education is a fundamental human right and every nation’s citizens should be granted universal access with equal quality to it. Because this goal is yet to be achieved in most countries, in particular in the developing and underdeveloped countries, it is extremely important to find more effective ways to improve education. This paper presents a model based on the application of computational intelligence (data mining and data science) that leads to the development of the student’s knowledge profile and that can help educators in their decision making for best orienting their students. This model also tries to establish key performance indicators to monitor objectives’ achievement within individual strategic planning assembled for each student. The model uses random forest for classification and prediction, graph description for data structure visualization and recommendation systems to present relevant information to stakeholders. The results presented were built based on the real dataset obtained from a Brazilian private k-9 (elementary school). The obtained results include correlations among key data, a model to predict student performance and recommendations that were generated for the stakeholders.

## 1. Introduction

Considered to be one of the most important needs of every citizen, modern societies classify education, which plays a key role in the development of any country [1], as a fundamental right of people. Throughout history, education faced a number of challenges and paradigm shifts. Nowadays, managers and institutions are concerned with retaining students and making learning interesting, efficient and effective. Parents, in turn, are committed to understanding children’s performance and helping them with learning. Teachers and educators want to understand the real situation of the teaching-learning process, with accurate information that might guide and create value through learning. Finally, students want to learn.

The dynamics of the teaching-learning process has faced rapid transformations promoted by the outstanding advances of Information and Communication Technologies (ICTs) and business models. According to Stuchlikova [2], the forecast is that knowledge doubles every three years. This means that much of the knowledge acquired during a degree, for example, will be obsolete one it is completed. The author also quotes some challenges that education is already facing, such as:

- Creative school, which should act differently for each student, seeking to fully develop him or her, as opposed to offering a standard curriculum.
- Self-learning promotion, with several initiatives to promote self-study, including the offering of courses for all ages and areas of knowledge.
- Virtual world and virtual teachers, with applications where the classroom breaks the boundaries of the school.

Gartner Inc. (Stanford, CT, USA) [3,4], in turn, exposes key business and technological trends that will influence fundamental and higher education, as shown in Table 1 and Table 2.

In face of the challenges and trends exposed above, Arold and Pistilli [5] describe that the key to success is integrating students with modern technological transformations and innovations. They explain that it is necessary to obtain integrated and accurate information (internal and external to the school) of students in order to provide parents, educators and school managers with the adequate understanding to support the decision-making process that shall direct the teaching and learning activities, which will probably be different for each student.

As an example of student integration with ICTs, Meneghel [6] emphasizes the increasing use of Virtual Learning Environments (VLE) and virtual Labs (vLabs) to improve Distance Education (DE). Based on these concepts and due to the high level of interaction between modern students and learning (including e-learning) objects, it is possible to construct an educational (big) database that allows the mining and recognition of educational patterns. Moreover, the system may acquire information that could be used to generate indicators that measure students’ performance.

Considering today’s diversity and extension of knowledge, it is important to develop solutions that can help educators in assessing student knowledge and automate decision-making processes [7]. In this case, educators must act as a kind of data science agent, directly or with the help of automated intelligent agents that act to perform tasks in response to some demand. These intelligent agents may be required to perceive their environment, persist over a prolonged time period, adapt to change and create and pursue goals. A rational agent is one that acts so as to achieve the best outcome or, when there is uncertainty, the best expected outcome [7].

It can be initially considered that all agents are interested in maximizing their outcomes. There are three fundamental agents in education: students, educators and administrators. Tasks that normally concern educational agents are, for example, identifying students’ profiles, predicting students’ performance, analyzing students’ social network interactions and planning and scheduling of courses.

This work addresses the decision-making process in educational by proposing and developing a model to describe students’ profiles and to develop a recommendation system to help students, educators and administrators to increase the odds of succeeding in their proposed educational goals. This model is based on an integration of a three-layered system: a web educational environment for distributing educational classes and games; a data collector and data mining system; and a data science platform for data processing and cognition extraction. This framework, which was named Edukas, has as its main goals: (i) create a real-time visualization environment to allow teachers and educational officials to identify the strengths and weaknesses of their teaching-learning process; (ii) establish a best practice guide for educational governance; (iii) use computational intelligence, learning analytics and data mining tools to create students’ learning profiles to help in students’ orientation; and (iv) identify and promote educational opportunities for each student based on his/her profile.

Finally, this work has been crafted with the overall idea of smart education in a smart city. Smart cities suppose cities with sensor nets, IoT, smart health, smart governments, smart buildings, smart cars, and so on. The goal here is to add a piece in the puzzle assembly.

This paper is organized as follows. This first section presents basic considerations and justifications for this work and defines its main objectives. Section 2 presents an overall review of the key topics treated here and the main works upon which this work is based. Section 3 presents the main concepts behind the Edukas environment and describes the proposed model. Section 4 presents the data structures used to characterize the subjects. Section 5 presents the results obtained from the data analysis and specifies recommendations to stakeholders. In the last section, the conclusions and future developments are presented.

## 2. The Conceptual View

In this section, key topics related to this research are presented. Due to paper size limitation, only summarized concepts and basic definitions are described.

### 2.1. Corporate and Educational Governance

Corporate Governance (CG) is the application of a management system in which organizations are directed, monitored and encouraged, taking into account the relationships between owners, directors, executive officers and other control bodies and employees where the best practices procedure are taken into consideration. Good CG practices convert principles into objectives, which, in turn, preserve resources, optimize organization’s value and facilitate access of external capital, contributing to corporation’s longevity [8]. CG plays a key role in today’s businesses. Its application often defines how the organization is accepted and recognized by society. Good CG practices are essential for the private sector and have led to economic growth and promotion of social welfare, which is largely dependent on increasing investments, capital market efficiency and companies’ performance [9].

Corporate governance considers what are the organization’s stakeholders’ needs, both internal and external, and what are their drivers. A driver is understood as someone, something or some situation (internal or external) that may orient an organization and its stakeholders in the direction of their needs, and these should be considered, addressed and used to direct the organization’s objectives.

In education, CG can be defined as the processes that guarantee the efficient and effective use of resources to enable the organization to achieve its overall goal of transmitting established knowledge to new generations along with the power to make this knowledge evolve. It is important to consider key education stakeholders, as well as their drivers. The following Table 3 shows examples identifying stakeholders, their drivers and needs.

In Table 3, the first column shows who is the interested stakeholders. The second column discloses what they want from education. Finally, the third column shows which drivers can influence their needs. For example, society is looking for well-formed and economically-productive citizens and is driven by job markets, individual and collective desires, society, the economy and technological trends. Information about stakeholders and their needs and drivers is not limited to those displayed in Table 3.

**Figure 1:**
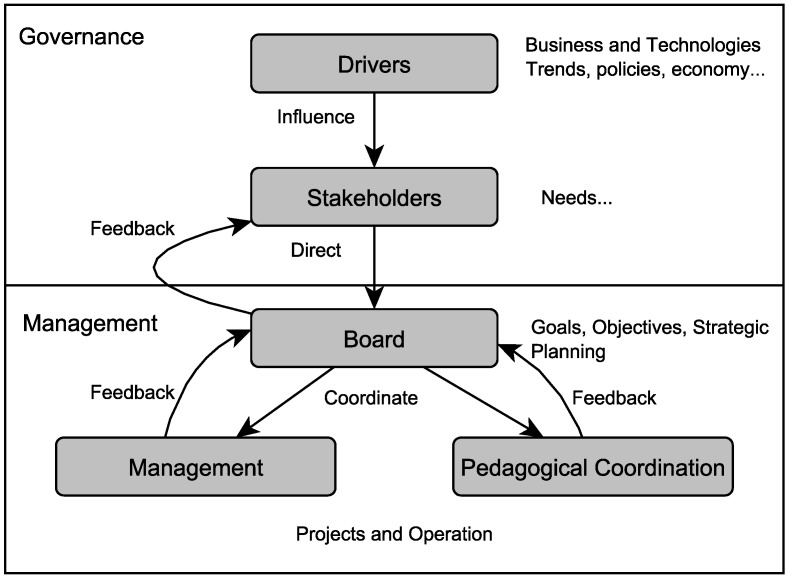
Macro-model of governance and educational management.

Stakeholders can be classified as direct or indirect. Direct education stakeholders are those who are directly involved in the teaching-learning activities, e.g., school managers, parents, teachers and, obviously, students. Indirect education stakeholders are those who do not have a direct involvement in the activities, but have expectations related to their results, e.g., government and society. Figure 1, below, presents the relationship between educational actors, their drivers and the management of an educational organization.

According to the macro-model presented in Figure 1, educational governance and management determine the relationships between stakeholders along with their drivers and the several levels of educational management. To simplify the figure, only two departments of a school were represented, management and pedagogical coordination. The name of these departments may vary from one school to another, but their functions are always present. For good educational governance, it is important that school’s structures and stakeholders are well-defined and always updated. For example, because of constant technological advances, the creation of an innovation department may be necessary.

The trends exposed in Table 1 and Table 2, alongside the drivers introduced in Table 3, influence stakeholders’ needs, which are strongly dynamic and time-varying.

In a corporation, the main objectives defined by the board must be interpreted by each management area, which should elaborate area objectives that should contribute to the accomplishment of those objectives. It is critical that every corporation department work together and help in the development of the corporation’s governance plan, translating it into action plans, projects and other activities. However, because education stakeholders’ needs are dynamic and time-varying, they may be difficult to identify, and it may be difficult to comprehend their full extension clearly. One important goal of the proposed model is to more accurately identify and comprehend stakeholders’ needs and to offer an environment where this comprehension can be transformed into actions to reach their needs.

An important characteristics of the Edukas model is that it can make parts of an analytical process transparent, allowing users to comprehend how procedures, like feedback, may influence the results. Thus, users may get insights on how to operate the system, from the most operational levels to the most strategic ones, in order to search for the aimed objectives. Furthermore, this process understanding allows for corrective actions to be taken as soon as possible.

### 2.2. Strategic Planning

The term “strategy” (from the Greek στρατηγία, “art of the general”) has its origins in the ancient civilizations and military endeavors. The adaptation of this term to business resulted in the “strategic planning” procedure. Acordding Drucker (1954) [10], the strategic planning activities give to managers the ability to arrange tasks, establish missions and determine the short-, medium- and long-term goals of an organization.

In the science of business, the last few decades were marked by intense discussions about the factors that should compose strategic planning [11,12,13], its responsibilities [14,15,16] and ways of developing, improving and monitoring the implementation of the plans [17,18,19,20].

Strategic planning is a process and thus must be characterized by inputs, activities, outputs and outcomes. There is a variety of tools and techniques developed to provide a framework for implementing strategic planning. The SWOT (Strengths, Weaknesses, Opportunities and Threats) analysis [11], for example, is an excellent tool to guarantee the equilibrium between internal and external environments. Porter’s five forces analysis [12] helps to determine the attractiveness of an organization and its products. Another tool is the Balanced Scorecard (BSC) [21] methodology used to measure the business performance through quantifiable and verifiable indicators, which are interrelated in a cause-effect strategic map.

In education, several successful experiences with strategic planning in higher education institutions have been reported. In this case, the performed activities must work to respond quickly and positively to the growing progress of expectations attributed to the teaching-learning process, maximizing the chances of maintaining a competitive and relevant institution in the face of market developments [22].

A successful example was reported in Turkey [23]. Under current legislation and as part of a quality management program, since 2006, public educational institutions need to develop and maintain strategic plans. As a consequence, public resources have become better managed, bringing several improvements in the social areas of the institutions. To keep the plans updated, the institutions promote annual events to review the objectives and ensure the continuous improvement of the plans.

Another case of success is the Cheng Kung National University in Taiwan, which aims to become one of the world’s 100 best universities, as reported by Chou and Li [1]. Their strategic planning framework allows gauging how much the BSC objectives are aligned with the institutional objectives, resulting in a strategic map with cause and effect relationships.

Distance education has also employed strategic planning methodologies on its organizational processes. Carr-Chellman [24] states that planning has great importance to an institution’s success. Moreover, it is necessary to incorporate good practices to understand the implicit values of the teaching-learning process.

In any of the above-mentioned environments, the design of strategic planning is a very complex task [25]. In areas of great challenges and uncertainties, such as in education, strategic planning may play a fundamental role for the institutions to achieve their goals. Therefore, educators must use strategic planning with data organization and mining tools to integrate administrative and academic data, investments in infrastructure and human resources, applied questionnaires, grades, absenteeism reports, interaction with virtual learning systems and other kinds of metrics [26] and produce the necessary cognition for educational organizations.

### 2.3. Computational Intelligence over Educational Data Mining, Warehousing and Analytics

Educational Data Mining (EDM) is an interdisciplinary research area that deals with the development of methods to explore data originating in education. EDM uses computational approaches to analyze educational data and generates knowledge and cognition to help the process of decision-making. This area exploits the use of statistical, machine-learning and data-mining algorithms to analyze the different types of educational data. The EDM process converts raw data coming from educational systems into useful information that could potentially have a great impact on educational research and practice. In the following subsections, some common tasks that might be addressed using computational intelligence algorithms in education [27,28] are presented and discussed.

Furthermore, according to Mehmood et al. [29], nowadays, the use of Internet of Things (IoT), big data, artificial intelligence and deep learning and High Performance Computing (HPC) as a process infrastructure to provide support for data analysis based on media and sentiment analysis of Massive Open Online Courses (MOOC) is of vital importance. As a result, the authors can predict situations such as the following:

1. To provide adaptive educational content delivery to students who are registered for a course.
2. To identify students based on the spatio-temporal activity data.
3. To use the obtained temporal activity patterns for computing and predicting the number and type of clients and applications.
4. To compute and predict the bandwidth, latency and other characteristics of the networks required by the system based on using the data obtained.
5. To compute and predict computational and other system characteristics required by the system by using the data obtained.

The Edukas model addresses the three first situations providing a recommendation system based on students’ trails and a probabilistic model (explained in Section . This model is used to improved the accuracy of recommendations. Besides, the work of Mehmood et al. focuses on infrastructure to provide an architecture to implement data analysis. The Edukas model is a computational model supported by drives obtained by the use of educational data generated in an educational and pedagogical environment with information feedback in several analytics tools, including governance and strategic planning tools built in response to the scenario met by the system.

#### 2.3.1. Classify Students Profiles

The objective of this task is to identify and suggest students’ profiles that provide useful information to help educators to understand the large variety of qualities and quantities that can be considered when trying to group students into categories of skills, fitness, interests, perceptions, etc. Classifiers such as random forest, decision tree and kNN are usually applied in this context. Moreover, a suitable profile might be a first step to recommend an adequate path for students that aim to achieve specific goals [27,28].

#### 2.3.2. Recommendations for Student

The objective here is to attend a student demand for specific results with recommendations such as personalized activities, adapted learning contents, suggested links to visit or the next task or problem to solve. Several data mining techniques can be used to address this task, but the most common are association rule mining, clustering and sequential pattern mining. Association rule mining is a procedure used to discover relations existent between variables defined in large databases. Clustering is a data mining technique to reach data grouping based on similarities. Sequence/sequential pattern mining can be used to discover relationships between occurrences of sequential events to find if there exists any specific order in which these occurrences should happen [28].

#### 2.3.3. Predicting Student Performance

The prediction of student performance is aimed at estimating the unknown value that a variable that describes the student will assume in the future. In education, the values normally predicted are performance, knowledge, score or mark. This value can be a numerical/continuous value (regression task) or a categorical/discrete value (classification task). Regression analysis finds the relationship between a dependent variable and one or more independent variables. Classification is a procedure in which individual items are placed into groups based on quantitative information regarding one or more of their characteristics and based on a training set of previously labeled items. Prediction of student performance is one of the oldest and most popular applications of data mining in education. Different techniques and models have been applied to accomplish such prediction such as neural networks, Bayesian networks, rule-based systems, regression and correlation analysis [27,28].

#### 2.3.4. Student Modeling

The objective of student modeling is to develop cognitive models of human users/students, including the modeling of their skills and declarative knowledge. Data mining has been applied to automatically consider user characteristics (motivation, satisfaction, learning styles, affective status, and so on) and learning behavior in order to automate the construction of student models. Different data mining techniques and algorithms have been used for this task (mainly, Bayesian networks) [28].

#### 2.3.5. Detecting Undesirable Student Behaviors

Undesirable student behavior can also be discovered/detected using data mining techniques. In this case, those students who present some type of problem or unusual behavior such as erroneous actions, low motivation, playing games, misuse, cheating, dropping out or academic failure, among others, can be analyzed and detected. Several techniques (mainly, classification and clustering) have been used to discover these types of behaviors. This can help educators to better understand and orient students who fall into this category [28].

#### 2.3.6. Grouping Students

To better understand their knowledge profiles, students can be grouped according to their customized features, personal characteristics, etc. The clusters/groups of students obtained can be used to define a set of tasks for orienting the students, such as the construction of personalized learning activities, the promotion of effective group learning in some areas, the provision of some set of adaptive contents, etc. The data mining techniques normally used in this tasks are classification (supervised learning) and clustering (unsupervised learning). Cluster analysis or clustering is the assignment of a subject with a set of observations into a subset (called a cluster) so that subjects with similar observations can be set to that same subset [27,28].

#### 2.3.7. Social Network Analysis

Social Network Analysis (SNA), or structural analysis, aims at studying relationships between individuals, instead of individuals’ attributes or properties. A social network is considered to be a group of people, individuals that initially can be either unrelated or pertaining to an organization, who are connected using social relationships such as friendship, cooperative relations or information exchange. Different data mining techniques have been used to mine social networks in educational environments, but collaborative filtering is the most common of them. Collaborative filtering or social filtering is a method of making automatic predictions (filtering) about the interests of a user by collecting and classifying taste preferences from many users (collaborating). Collaborative filtering systems can produce personal recommendations by computing the similarity between students’ preferences, thus making this task directly related to the previous task of recommendations for students [27].

#### 2.3.8. Providing Feedback for Instructors

One of the most important reasons to develop intelligent systems in education is to provide feedback to support course authors, teachers and administrators in their everyday decision-making responsibilities. Tasks such as how to improve students’ learning, organize instructional resources more efficiently and enable students to take appropriate proactive and/or remedial action are of primal concern to teachers and administrators. Intelligent systems can substantially improve the rate of success in these cases.

It is important to point out that providing this feedback is different from data analyzing and visualizing tasks, which only provide basic information directly from data (reports, statistics, etc.). Moreover, this procedure uncovers completely new and interesting information existent in the data, but that can usually be hidden behind the normally huge amount of information available. Several data mining techniques have been used to accomplish the feedback to instructors, although association-rule mining has been the most common. Association-rule mining reveals interesting relationships among variables in large databases and presents them in the form of strong rules according to the different degrees of interest they might have [27].

#### 2.3.9. Developing Concept Maps

A concept map is a conceptual graph that determines relationships between concepts and subjects and presents them in a graphical map creating an hierarchical structure of knowledge. Concept maps offer an excellent tool to help instructors/educators in acquiring the knowledge obtained with some of the data mining and data analysis described above by visualizing it through a conceptual diagram. The automatic process of developing/constructing concept maps is a required tool for any intelligent system. There are several data mining techniques (mainly, association rules and text mining) that can be used in the construction of concept maps [28].

#### 2.3.10. Constructing Courseware

Because constructing/adapting courseware is one of the main works teachers, and educators in general, are concerned with, it is extremely important to offer tools that help in the automation process of producing learning materials. These tools can also offer the benefit of promoting the reuse/exchange of existing learning resources among different users and systems [27,28].

#### 2.3.11. Planning and Scheduling

The objective of planning and scheduling is to enhance the traditional educational process by planning future courses, helping with student course scheduling, planning resource allocation, helping in the admission and counseling processes, developing curriculum, etc. Planning and scheduling can offer information that can be used by data analysis to orient decision-making based on several data mining techniques (mainly association rules).

#### 2.3.12. Analysis and Visualization of Data

The objective of the analysis and visualization of data is to highlight useful information and support decision-making. In the educational environment, visualization can help educators and course administrators to analyze students’ course activities and get a general view of a student’s learning development. Statistics and visualization information are the two main techniques that have been used to accomplish this task. Information visualization uses graphic techniques to help people understand and analyze data. Visual representations and interaction techniques take advantage of human eyes’ broad bandwidth pathway into the mind to allow users to see, explore, and understand large amounts of information at once [27,28].

#### 2.3.13. Stakeholders Point of View

**Figure 2:**
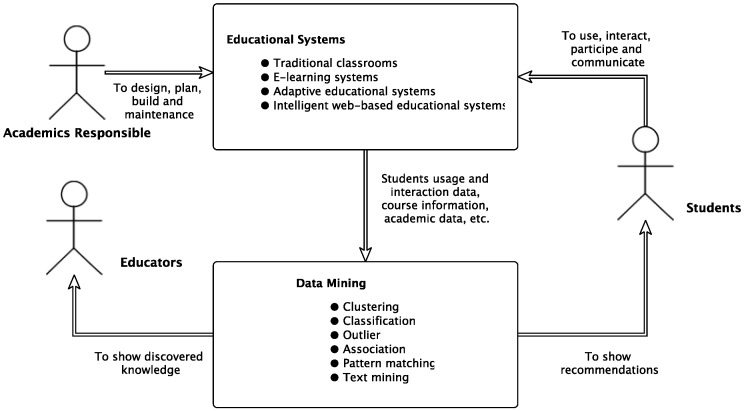
An overview of a unified educational system [27,28].

The idea here is to allow a specialized view of the analyzed results for different kinds of stakeholders. Despite these different views, all information might be provided by a single unified system. Figure 2 below shows an overview of a unified educational system.

As shown in Figure 2 above, a unified educational system must carry three different points of view:

- Oriented toward students: The student view creates the need for specific recommendations for learners such as: activities, resources and learning tasks that would favor and improve their learning; catalog and classify history of good learning experiences from other students; path pruning and shortening or simply links to follow, based on the tasks already done by the learner and/or other learners; etc.
- Oriented toward educators: Educators’ goals are to get more objective feedback for instruction, evaluate the structure of the course content and its effectiveness on the learning process, classify learners into groups based on their needs in guidance and monitoring, find learners regular as well as irregular patterns, find the most frequently made mistakes, find activities that are more effective, discover information to improve the adaptation and customization of the courses, restructure sites to better personalize courseware, organize the contents in a format that is more suitable for a specific student or group of students (this suitability should be discovered beforehand), help students to adaptively assemble their instructional plans, etc.
- Oriented toward academic administrators: Academic administrators want to obtain information on: how to improve site efficiency, how to adapt the sites to the behavior of their users (optimal server size, network traffic distribution, etc.), how to better organize institutional resources (human and material) and make them available to students, how to enhance educational programs’ offer and determine the effectiveness of the new computer-mediated distance learning approach, etc.

In short, educational data mining is a young research field, but is already recognized as of utmost importance to allow the development of more specialized and oriented work in education. It is expected that EDM can obtain a similar application success level as data mining has obtained in other areas, such as medical data mining, mining e-commerce data, etc.

As shown in Table 4, some common educational tasks may support research in computational intelligence, thus providing useful information that may be used by decision-makers to improve the quality of general education.

### 2.4. Human Decision-Making

Human decision-making is a cognitive process that has been studied and widely applied in many disciplines encompassing psychology, neuroscience, cognitive computation, computer science, management science, economics and statistics, among others.

There is evidence showing that decision-making is a cognitive function developed in the Prefrontal Cortex (PFC) of the brain. According to Cervantes [30], the Orbitofrontal Cortex (OFC) is unique in receiving information from all sensory modalities: visual, olfactory, gustatory, auditory and somatosensory. It also receives motivational and emotional information. These brain areas offer highly processed information about the environment to the decision-making process. This information is both internal and external. Generally speaking, this process is similar to an ant colony that marks the path from the nest to food sources and back to the nest. Therefore, it is important to look for brain and ant colony models in order to build automated decision-making models for computational intelligent systems.

### 2.5. Ant Colony

Ant colonies, and more generally, social insect societies, are distributed systems that, in spite of the simplicity of their individuals, present a highly structured social organization. As a result of this organization, ant colonies can accomplish complex tasks that, in some cases, far exceed the individual capabilities of a single individual. Ants coordinate their activities via stigmergy, a form of indirect communication mediated by modifications of the environment. In other words, biologists have shown that it is often sufficient to consider stigmergic indirect communication to explain how social insects can achieve self-organization.

### 2.6. Graphs

A graph is an ordered pair G=(V,E) comprising a set *V* of vertices or nodes or points together with a set *E* of edges or arcs or lines, which are two-element subsets of *V* (i.e., an edge is associated with two vertices, and that association takes the form of the unordered pair comprising those two vertices). To avoid ambiguity, this type of graph may be described precisely as undirected and simple.

*V* and *E* are usually taken to be finite, and many of the well-known results are not true (or are rather different) for infinite graphs because many of the arguments fail in the infinite case. The order of a graph is |*V*|, its number of vertices. The size of a graph is |E|, its number of edges. The degree or valence of a vertex is the number of edges that connect to it, where an edge that connects a vertex to itself (a loop) is counted twice.

## 3. Model

This section presents the basic elements of the Edukas environment, showing how this work treats the link between educational governance, strategic planning and computational intelligence. The Edukas model will be focused in of of the tasks mentioned above: (1) classify students’ profiles, (2) recommendations for students and others and (3) predicting student performance. This section is finalized with the presentation and discussion of the paper hypothesis.

### 3.1. Edukas Environment

**Figure 3:**
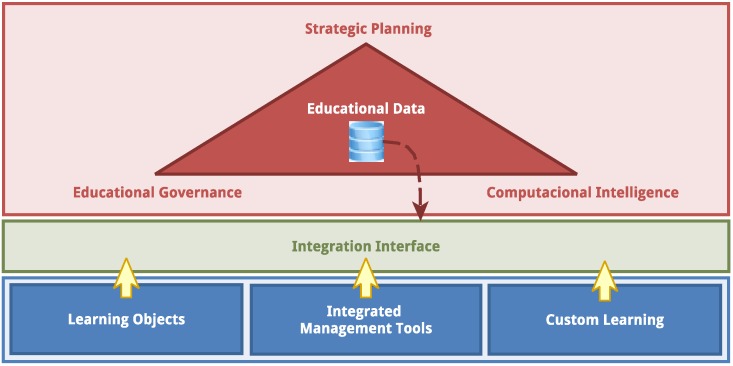
Edukas environment conceptual model.

Based on the exposed background, the Edukas environment comprises three main layers, as shown in Figure 3. The lowest layer contains the systems that can be accessed by the stakeholders. The middle layer records all data resulting from the interaction with these systems, forming a educational database. The highest layer uses the data stored in the database, transformed by the corporate governance model, by the computational intelligence algorithms and by the strategic planning tools, to generate tangible information for the stakeholders.

The lower layer is responsible for the collection and structure of data. It is in this layer that the administrative, academic and content systems are deployed and used to generate the transactional information of the educational communities. The middle layer is responsible for housing data mining and data preparation tools. Finally, the upper layer is responsible for the data analytics and the decision-making, which leads to the strategic planning, to the educational governance and to the orientation tools used by educators.

Data collected in the lower layer can be classified into three groups, depending on their origin: learning objects, integrated management tools and custom learning. Data from the learning objects are generated from digital activities, mainly educational games and digital courseware, that are submitted to students to present knowledge and content and to evaluate students’ performances. The learning objects use different technologies such as artificial intelligence, virtual/augmented reality, digital assessment, adaptive learning, listening and sensing technology and robotic telepresence. Learning objects’ data allows the definition of students’ knowledge profiles, which is a history map showing the knowledge each student has built up to some point in time.

Data from integrated management gathers information from heterogeneous data provided by different education management systems. These are mainly administrative data.

Data from custom learning use the teaching-learning activities to identify attributes for each student and discover the students’ learning profiles, which are history maps that show how students work in order to acquire their knowledge. These maps can help educators to identify and solve gaps in the teaching-learning process. The creation of the learning profiles uses technologies such as adaptive learning, artificial intelligence, digital assessment, listening and sensing technology, predictive analytics and hybrid integration platforms.

In the upper layer, strategic planning provides the direction to reach the goals; educational governance establishes the goals and monitors if the organization reaches them or not; and computational intelligence provides real-time analysis of information for the decision-making. These three tools must be tightly related and integrated. They make use of the educational database that collects data of different types and from different sources.

Edukas is, at the same time, a learning environment, a management system and an analytics framework, which are defined by each of the three layers discussed above. With Edukas, students receive education content from the learning environment; data scientists gather the vital information to analyze each student and improve the odds for him/her to obtain the best education the system has to offer. With the Edukas framework, educators can use assessment methods and define road maps to define actions that can lead to the best decision-making processes.

The discussion presented so far, and considering that the driver “good student performance” leads to the objective “improve the student success rate”, as shown in Table 5, shows that modern education cannot afford being left out of the “smart” movement that is taken over most of the science and business areas of contemporary society.

Now, it is necessary to define how to develop the analysis that can generate the indicators composing the students’ profiles discussed before. These indicators are described in Section 5.

### 3.2. Hypothesis

**Figure 4:**
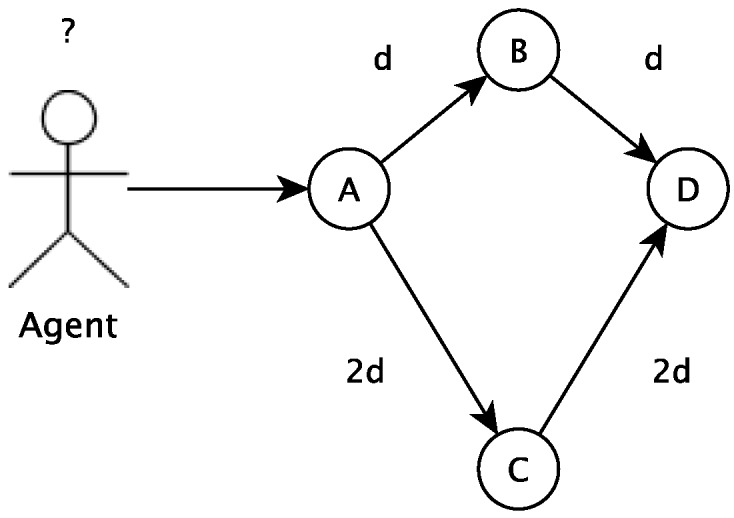
An overview of the main hypothesis of this work. Branches have different lengths.

In this section, the algorithm used to choose a path, among several others, is presented. This algorithm is based on the ant colony concept. The algorithm tries to define the best path to follow based on past utilization results of all paths. In the proposed algorithm, a vertex describes a particular state of an agent (which can be a student, a group of students, an educator, etc.). To go from one state to another, the agent has to undergo a transition, defined by a path in the algorithm. A procedure (a student playing an educational game, for example) can move the agent between states. The agent movement can be modeled in a graph G=(V,E) where each vertex (V) is a possible state to where the agent can move and the edges (E) connecting the states are the possible paths to navigate from one vertex (a state) to another, as shown in Figure 4.

In the beginning, an agent initial state must be defined. If this agent is a student, for example, its initial state can be defined by classification and clusterization tasks. The initial state is a vertex in the graph. The other vertices are different states the student can go to departing from the current state. There are vertices (states) that are not directly connected to the current vertices, and the student has to go to more than one change of state to get there. The recommendation tool will calculate the cost (for example, number of study hours, number of disciplines or related activities) associated with all possible paths. Based on the ant colony concept, the recommended next vertex (or state) will consider previous students’ chosen next state.

There is a cost associated with the movement from one state to another; then, paths have costs associated with them. This cost might be a function or many functions f(x), which represent how easy or difficult it is to follow that path. Moreover, there is a set of information that is used to decide about the next vertex. This information might be used to represent if the agent is predisposed to follow the path modeled. This predisposition or tendency can be modeled by a function f(y). Therefore, the total cost is defined as f(t)=f(x)+f(y). The two main hypotheses of this work are:

**Hypothesis** **1.** The agent (or decision-maker) uses cost and predisposed information to decide which is the next path to choose. Predisposed information might be defined as a pheromone.

**Hypothesis** **2.** The agent behavior (or decisions) might be predicted using the current state, paths, cost functions and pheromone functions.

Some assumptions were considered to improve the model quality:

- Humans are social agents; thus, they exchange messages based on the pheromone (information)
- When an agent decides and acts, a trace of the pheromone is created for each path used by this agent
- Each agent tends to choose a path with the greater concentration of pheromone
- The vertex *A* represents initial context, and vertex *D* represents final context (or goal)
- The graph is considered a Markov chain

To find each agent’s real state, agents can initially be put on randomly chosen vertices. At each construction step, agent *k* applies a probabilistic action choice rule, called the random proportional rule, to decide which vertex to visit next. In particular, the probability with which agent *k*, currently at vertex *i*, chooses to go to vertex *j* is [31]:

(1) $$\begin{array}{c} p_{ij}^{k}\left(t\right)=\frac{{\left[\tau_{ij}\left(t\right)\right]}^{\alpha }\cdot {\left[\eta_{ij}\right]}^{\beta }}{\sum l\in N_{i}^{k}{\left[\tau_{il}\left(t\right)\right]}^{\alpha }\cdot {\left[\eta_{il}\right]}^{\beta }},ifj\in N_{i}^{k} \end{array}$$

where $\eta_{ij}=1/d_{ij}$ is a heuristic value that is available a priori, *a* and *b* are two parameters that determine the relative influence of the pheromone trail and the heuristic information and $N_{i}^{k}$ is the feasible neighborhood of agent *k* when being at vertex *i*, that is the set of vertices that agent *k* has not visited yet (the probability of choosing a vertex outside $N_{i}^{k}$ is zero).

By this probabilistic rule, the probability of choosing a particular arc (i,j) increases with the value of the associated pheromone trail $\tau_{ij}$ and of the heuristic information value $\eta_{ij}$.

**Figure 5:**
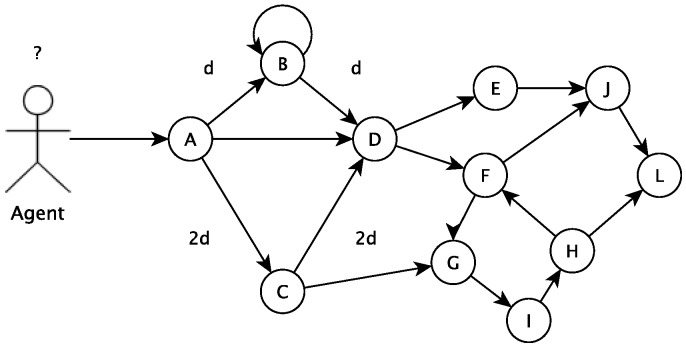
Example of the tour.

The role of the parameters α and β is the following. If α=0, the closest vertex is more likely to be selected. This corresponds to a classic stochastic greedy algorithm with multiple starting points since ants are initially randomly distributed over the cities. If β=0, only pheromone amplification is at work, that is only the pheromone is used, without any heuristic bias. This generally leads to rather poor results, and in particular, for values of α>0, it leads to the rapid emergence of a stagnation situation, a situation in which all the agents follow the same path and construct the same tour, which, in general, is strongly sub-optimal. Figure 5 below shows an example of this tour.

After all the agents have constructed their tours, the pheromone trails are updated. This is done by first lowering the pheromone value on all arcs by a constant factor and then adding pheromone on the arcs the ants have crossed in their tours. Pheromone evaporation is implemented by:

(2) $$\begin{array}{c} p_{jt}^{k}\left(t\right)=\tau_{ij}\left(t+1\right)=\left(1-\rho \right)\cdot \tau_{ij}\left(t\right)+\rho \cdot \Delta_{ij}^{best}\left(t\right) \end{array}$$

where 0<ρ<1 is the pheromone evaporation rate. The parameter r is used to avoid unlimited accumulation of the pheromone trails, and it enables the algorithm to “forget” bad decisions previously made. In fact, if an arc is not chosen by the agents, its associated pheromone value decreases exponentially in the number of iterations. After evaporation, all agents deposit pheromone on the arcs they have crossed in their tour, which is calculated by:

(3) $$\begin{array}{c} \tau_{ij}=\tau_{ij}+\sum_{k=1}^{m}\Delta_{ij}^{k},\forall \left(i,j\right)\in L \end{array}$$

where $\Delta_{ij}^{k}$ is the amount of pheromone ant *k* deposits on the arcs it has visited, which is defined as:

(4) $$\begin{array}{c} \Delta_{ij}^{k}=\left\{\begin{array}{cc} 1/C^{k} & \;\mathrm{if}\;\mathrm{arc}\left(i,j\right)\;\mathrm{belongs}\;\mathrm{to}\;T^{k}\\ 0 & \;\mathrm{otherwise} \end{array}\right. \end{array}$$

In this last equation $C^{k}$, the length of the tour $T^{k}$ built by the *k*-th agent is computed as the sum of the lengths of the arcs belonging to $T^{k}$. By means of Equation (4), the better an agent’s tour is, the more the amount of pheromone the arcs belonging to that tour receive. In general, arcs that are used by many agents and which are part of short tours receive more pheromone and are, consequently, more likely to be chosen by ants in future iterations of the algorithm.

According to Dorigo [31], there are some aspects that should be considered when building an ant colony optimization model:

- Construction of the graph, to express the paths along the time that can be used;
- Constraints, to limit the path options in the graph;
- Pheromone trails, to define the strategy of pheromone deposit;
- Heuristic information, the functions that should be optimized;
- Solution construction, issues about software engineering;
- Pheromone update, the strategy to eliminate unused paths;
- Local search, the strategy to avoid premature optimization;
- Particularities, some specific point to be considered;
- Results, results in a simple means of expression;
- Remarks, points to express much information;

### 3.3. Problem Representation

Let us consider the minimization problem (S,f,Ω), where *S* is the set of candidate solutions, *f* is the objective function that assigns to each candidate solution s∈S an objective function (cost) value f(s,t)= and Ω is a set of constraints. The goal is to find a globally optimal solution $s_{opt}\in S$, which is a minimum cost solution that satisfies the constraints Ω.

The problem representation of a combinatorial optimization problem (S,f,Ω) that is exploited by the ants can be characterized as follows:

- A finite set $C=c_{1},c_{2},\;\cdots ,c_{N_{C}}$ of components is given.
- The states of the problem are defined in terms of sequences $x=[c_{i},c_{j},\;\cdots ,c_{k}]$ over the elements of *C*. The set of all possible sequences is denoted by *S*. The length of a sequence *x*, that is the number of components in the sequence, is expressed by |x|.
- The finite set of constraints Ω defines the set of feasible states χ, with $\tilde{\chi }\subseteq \chi$.
- A cost f(s,t) is associated with each candidate solution s∈S.
- In some cases, a cost, or the estimation of a cost, $J(x_{i},t)$, can be associated with states other than solutions. If $x_{j}$ can be obtained by adding solution components to a state $x_{i}$, then $J\left(x_{i},t\right)\leq J\left(x_{j},t\right)$. Note that J(s,t)=f(s,t).

## 4. Materials and Methods

**Figure 6:**
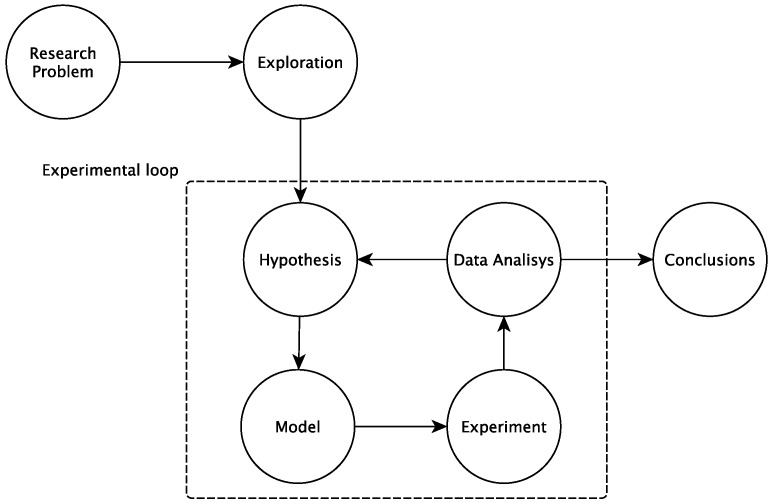
The process used in this work to discover an agent state.

The process used in this work to discover an agent state is shown in Figure 6 below. After identifying the research problem and defining the hypotheses and the model, experiments, based on real data, were performed to support preliminary data analysis and conclusions.

The figure above shows that a loop, called the “experimental loop”, is used to provide feedback and improve the accuracy of the hypotheses and model.

### 4.1. Datasets

The dataset used was collected from a Brazilian private school, with classes ranging from the second grade to the sixth, and refers to the 2017 calendar year results (with students from 7 and 11 years old). This school has the following characteristics (Table 6),

The school chosen for this study is considered to have a good level of quality. The dataset is composed by 48 attributes and 189 observations. Table 7 shows these attributes and types.

The attributes “target” are the expected values, and the attributes “qualifiers” are the quality from each observation of the dataset. The model was built by comparing qualifiers and target.

### 4.2. Algorithms

The algorithms used are based on Ant Colony Optimization (ACO) [31], Random Forest [32] (RF) and Recommendation Systems (RS) [7]. The ACO represents a significant step toward the definition of an efficient algorithm for the solution of minimum cost problems on graphs based on q stochastic mechanism and able to face NP-hard problems [31]. A random forest is a classifier consisting of a collection of tree-structured classifiers, which are independent identically distributed random vectors, and each tree casts a unit vote for the most popular class at a determined input [32]. The basic principle of recommendations is that significant dependencies exist between user- and item-centric activity [33]. In this context, one used RF to classify a student (agent) and RS to build recommendations to stakeholders over a graph model supported by ACO.

## 5. Results

### 5.1. Data Analysis

**Figure 7:**
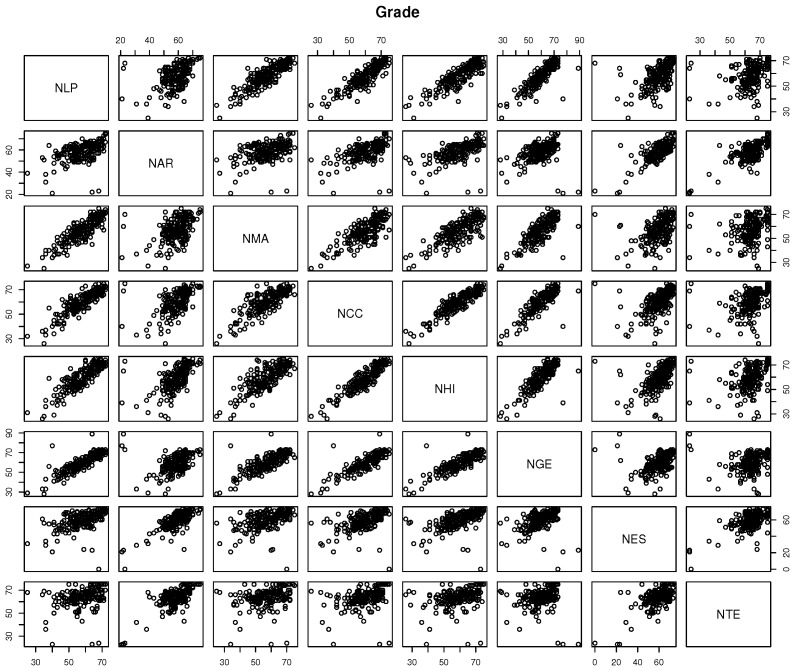
Correlations between disciplines and the associated grades. The disciplines’ grades are Portuguese language (NLP), Arts (NAR), Math (NMA), Science (NCC), History (NHI), Geography (NGE), Spanish (NES) and Technology (NTE).

Initially, the correlations between attributes must be identified. Figure 7 below shows the correlation between the disciplines and the associated grades obtained by the students.

As shown in Figure 7, there is a correlation between disciplines and grades. Now, it is possible to procure other correlations that allow the explanations for these results. For example, when a student scores high in some discipline, is it expected that he or she also gets high grades in some other set of disciplines? If so, can this common wisdom be verified?

**Figure 8:**
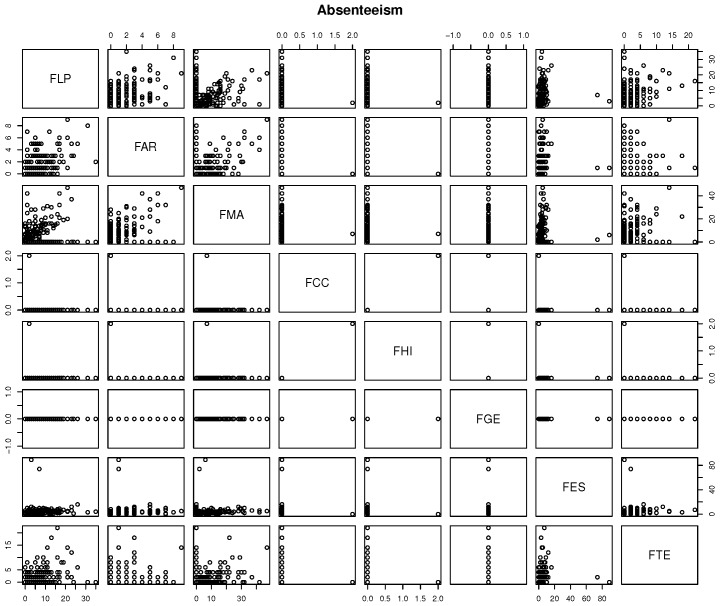
The correlation between discipline and students’ absenteeism. The disciplines’ absenteeism are Portuguese (FLP), Arts (FAR), Math (FMA), Science (FCC), History (FHI), Geography (FGE), Spanish (FES) and Technology (FTE).

Figure 8 shows that there is low correlation between absenteeism and some specific discipline. Thus, one cannot state that absentees in some discipline affects others.

**Figure 9:**
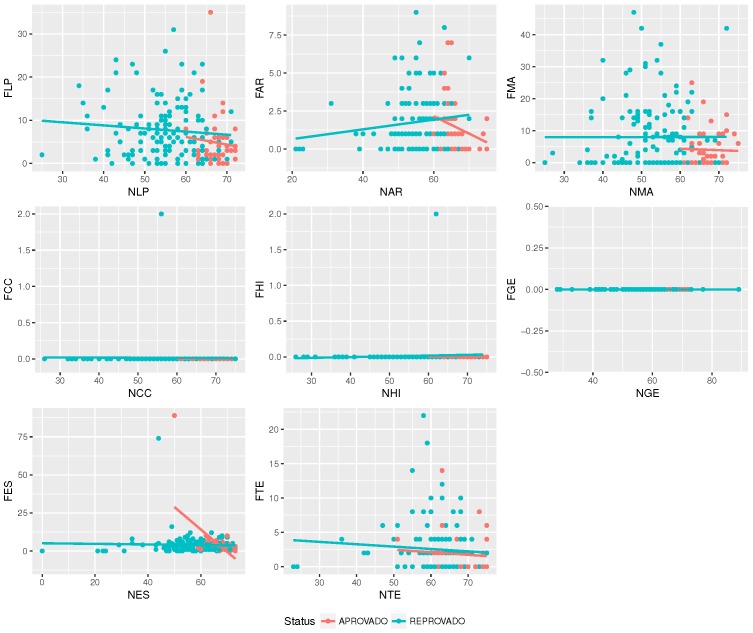
Correlation between discipline’s grade and absenteeism.

On the other hand, Figure 9 shows that there is a correlation between grade and absenteeism. It is possible to verify that less student absenteeism leads to higher students’ grades.

**Figure 10:**
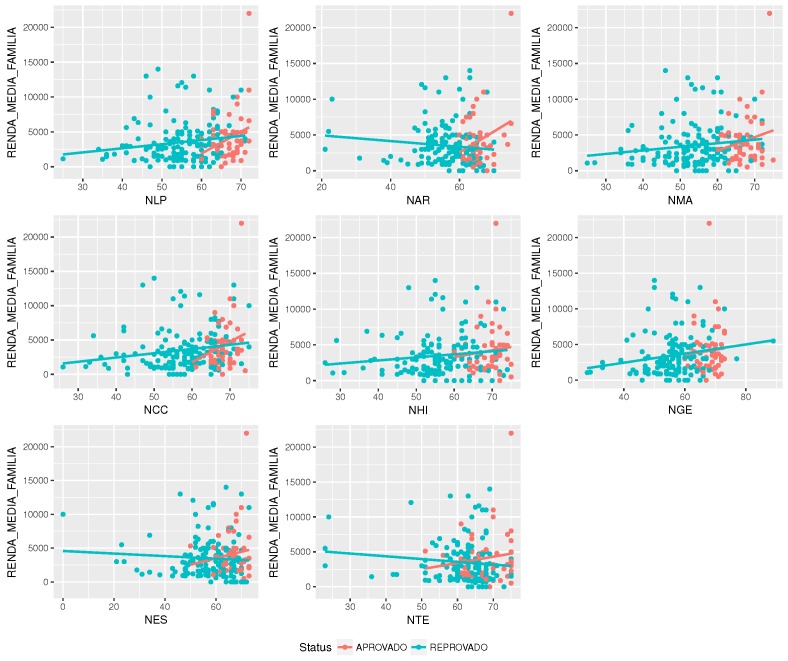
Correlation between discipline’s grade and income.

Families’ income is another variable that can be explored in terms of correlation with grades. Figure 10 suggests that there is a correlation between income and grade. Higher family income converts into higher grades. This correlation might be a consequence of a chain of correlations. For example, high income derives from profession, and profession derives from education. Thus, other variables may be more fundamental and can be controlling this results.

**Figure 11:**
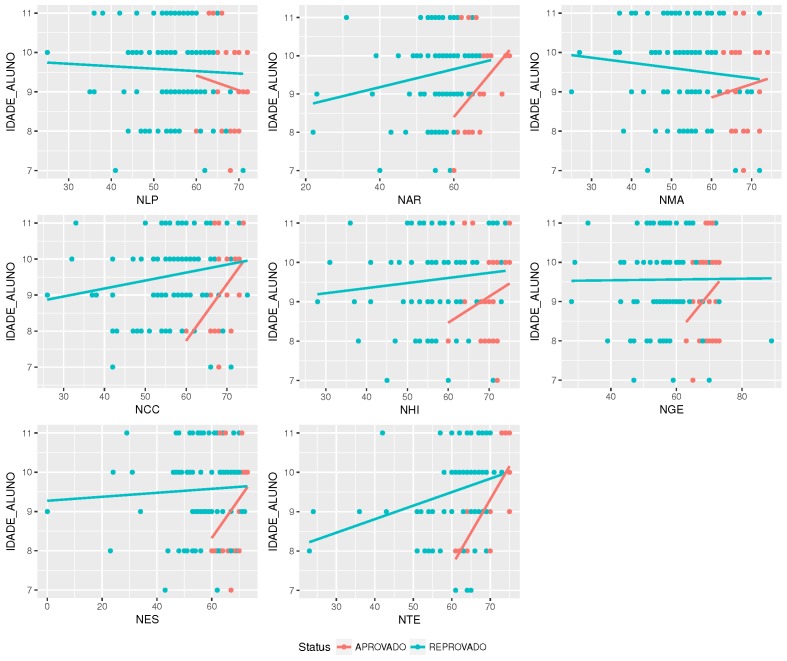
The correlation between discipline’s grade and student’s age.

Is there a correlation between age and grades? As shown in Figure 11, there is a correlation between age and grade. This case may be indicating that some content may not be adequate for some students’ ages.

**Figure 12:**
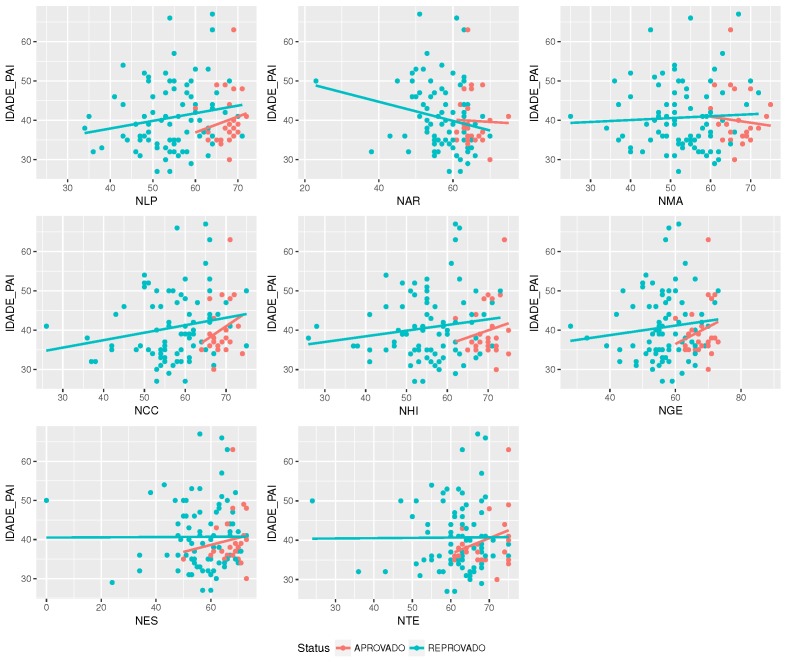
The correlation between discipline’s grade and father’s age.

Other kinds of correlations can also be pursued. Figure 12 shows that there is a correlation between father’s age and students’ grades. In this case, to determine the causes, it might be necessary identify the context of each age, for example income, financial stability, time available, emotional stability, among others. This correlation might express to administrators how to proceed with parents (or guardians) to help their students to improve their performance.

**Figure 13:**
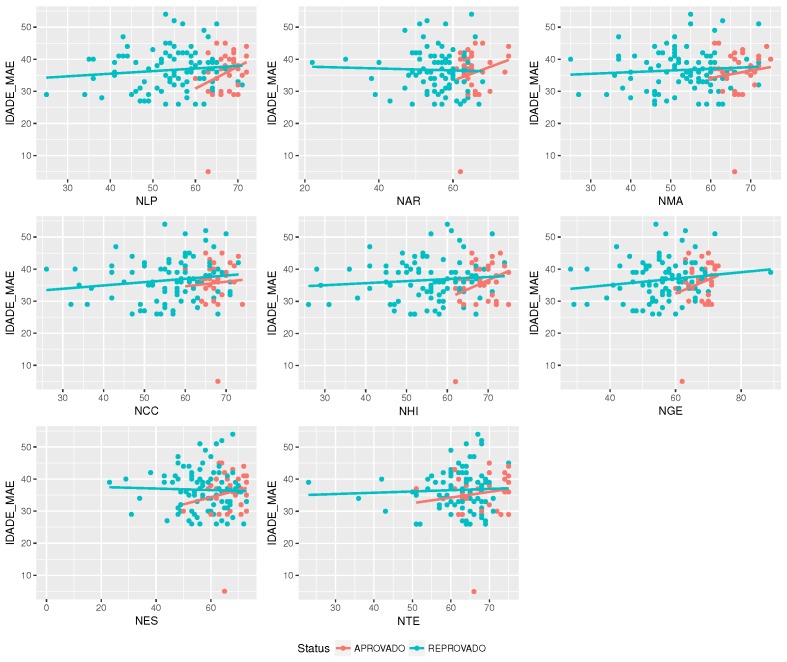
The correlation between discipline’s grade and mother’s age.

Equally, Figure 13 shows that there is a correlation between mother’s age and grade.

**Figure 14:**
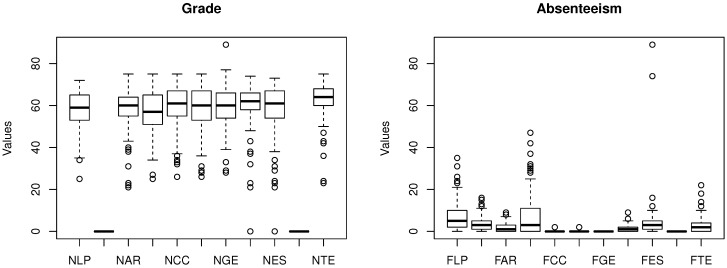
The variation of grade and absenteeism.

Figure 14 shows how grades and absenteeism values vary. As can be seen, grades vary from 0–100 and present the mean around 60. The promotion grade is 60, and in this case, several students fail to be promoted. The absenteeism ranges from 0–80 with the mean around five. The results do not permit identifying if absenteeism interferes with the results. An important factor to note is that, above grade 60, students tend to show a similar behavior. It is possible to notice a similar behavior on absenteeism, but in the opposite direction.

**Figure 15:**
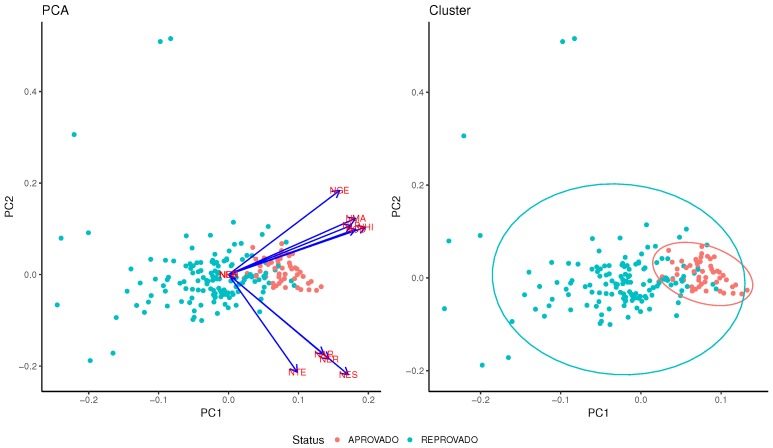
The PCA and cluster analysis.

Principal Component Analysis (PCA) consists of reducing attributes’ overlap. This can be used to identify if some attribute can be more relevant than the others. As can be seen from Figure 15, all continuum attributes that represent a discipline’s students’ grades are in the same direction, which means that none of them is more relevant than the others for the classification of a student as pass or fail. This means that there is no preponderant discipline that alone is capable of affecting the general results. A cluster analysis shows that the variation within the fail group (in blue) is higher than that within the pass group (red). This analysis was performed with grades of all disciplines.

**Figure 16:**
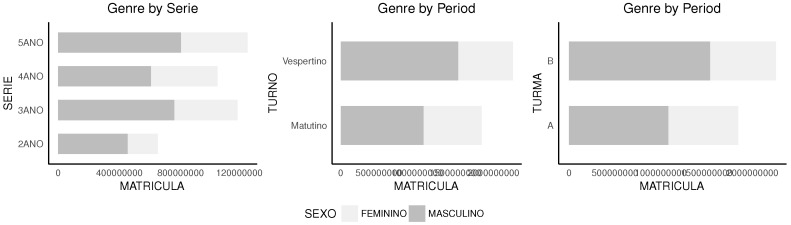
The distribution on the internal organization.

Finally, Figure 16 shows the distribution of genre over the dataset used. Despite not being explored in this work, genre distribution may have an important impact on how results may behave under different conditions.

### 5.2. Model

Some additional explanations must be given with respect to the conditions of the results obtained. The decision tree was grown using the qualifiers from students (TURMA, SERIE, SEXO, IDADE_ALUNO, IDADE_PAI, EDUCACAO_PAI, PROFISSAO_PAI, IDADE_MAE, EDUCACAO_MAE, PROFISSAO_MAE, RENDA_MEDIA_FAMILIA, CATEGORIA_ALUNO, TURNO). The qualifiers (MATRICULA, DATA_NASCIMENTO, BAIRRO, CIDADE, ESTADO, PAIS, CEP, DN, DN1) were suppressed because they do not affect the model. The class used was qualifier Status, which represents pass or fail in a general way, independent of the disciplines.

**Figure 17:**
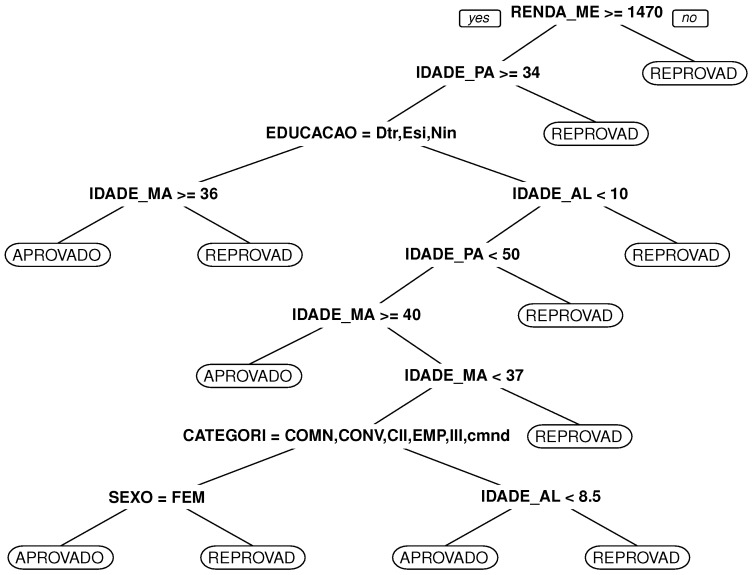
Tree grown using qualifiers and results.

As shown in Figure 17, the node RENDA_MEDIA_FAMILIA was the main node of the tree and represents the major factor that contributes to the results (pass or fail).

Using the model shown in Figure 17, a student (agent) can be classified, and his position in the graph model can be predicted, as explained in Section 3.2.

**Figure 18:**
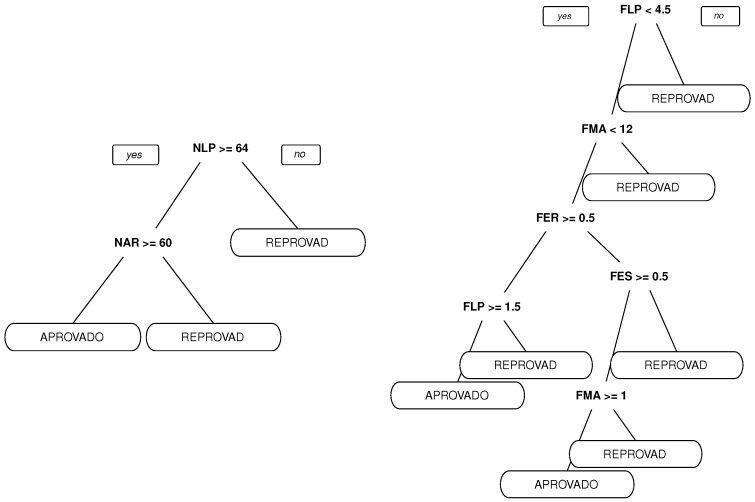
The tree grown using grade and absenteeism and results.

As shown in Figure 18, language grades and absenteeism are preponderant factors for the results.

**Figure 19:**
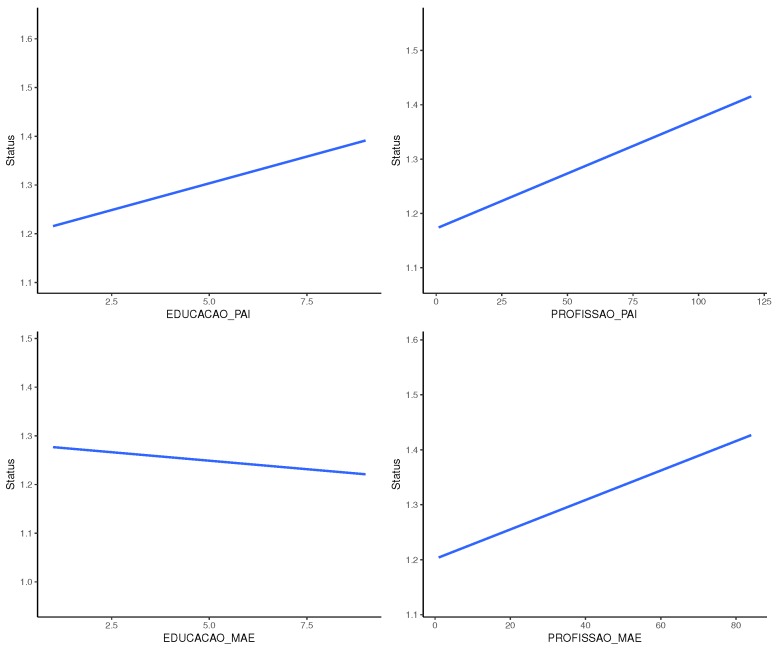
The correlation between father and mother’s education and profession and results.

Furthermore, Figure 19 shows that there is a correlation between parents’ education and profession and the obtained results.

### 5.3. Recommendations

One of the main goals of this project is to be able to create, based on the obtained results, a recommendation (or decision) map for stakeholders. This map should respond to each particular view or interest a stakeholder has in the education context.

- Students
  **Less** **absenteeism**. Based on their experience, teachers would call this an obvious recommendation, but results suggest that students should avoid missing classes in order to improve their results, as shown in Figure 9.
  **Study** **languages.** The results suggest that the study of language, in this case Portuguese, is a preponderant factor to achieve better results, as shown in Figure 18.
- Administrators
  **Income** **and grade are related.** The results recognize, in the cases analyzed, that better social and economic conditions might positively affect students’ results, as shown in Figure 10 and Figure 17. Here, education administrators should look for situations where good students’ results should not be related to parents incomes.
  **Father’s** **age and grade are related.** The results shown in Figure 12 are the kind of results that should be better understood. Administrators should look for conditions under which students’ performance should not be affected by factors external to school. The same can be said for the next four results.
  **Mother’s** **age and grade are related.** Idem.
  **Profession’s** **father and grade are related.** Idem.
  **Profession’s** **mother and grade are related.** Idem.
  **Education’s** **father and grade are related.** Idem.
  **Causes** **of pass and fail.** Identification of these causes is the desire of the main goals of projects like this, but the answers are not simple. A better approach, which this work expects to accomplish, is to offer on the fly suggestions for students in order to help them reach the pass condition with much higher probabilities.
- Educators
  **Grades** **in all disciplines are related.** At this level, it is common wisdom that students are more or less capable of equally handling most of the subjects. Thus, the result suggesting that good or bad results are independent of the subject, as shown in Figure 14, is not surprising. The main recommendation related to these results would be to observe students’ trends throughout the educational process.
  **Grade** **and absenteeism are related.** These results, as shown in Figure 10, suggest that absenteeism may have some interference with learning. Procedures to minimize or compensate absenteeism may help improve students’ performances.
  **Study** **languages.** The results shown in Figure 18 suggest that work in language (Portuguese in this case) may help students in any subject.
- Relatives
  **Less** **absenteeism.** Parents can have an important impact in preventing students’ absenteeism.
  **Study** **languages.** Reading programs with parents’ participation is recognized as an effective approach to help students develop the joy of learning.

It must be pointed out that the results presented here were discussed as a proof of concept for the Edukas project. To be able to make trustworthy assertions, one would need much more data and a much more carefully stated test condition. In any case, the main objective from such a process is to find ways to improve stakeholders’ performances and, consequently, improve the quality of education.

Finally, in order to accomplish this analysis, the available dataset allowed the definition of some performance indicators, which are decisive for the stakeholders to reach both the defined drivers and goals presented in Table 5.

## 6. Conclusions

As one of the several outcomes of this project, it is important to highlight the large support to stakeholders throughout the decision-making process. The project process allows the identification of the educational governance drivers and objectives along with the key performance indicators for their achievement.

The entire model is under a PDCA (Plan, Do, Check and Act) cycle. For each iteration, the results are analyzed and used to improve the model. Computational intelligence was used as a tool to determine the best indicators for each driver and goal.

A computational model was built to generate students’ profiles, which would allow better conditions for classifying students within pass/fail groups. The model can also be used to predict students’ behavior based on qualifiers analyzed in the dataset. Furthermore, many correlations between continuous and categorical variables can be used to look for information about these correlations’ cause.

After applying the model and obtaining the results, some recommendations for students, educators, administrators and relatives were developed as a proof of concept of this project application. In the ideal case, these recommendations should be used to identify indicators that lead to actions to improve stakeholders’ performance.

Further work will include applying the model to more controlled cases and investigating the model’s structure elements like cost functions f(t)=f(x)+f(y) and classifiers. Moreover, real-time decision-making is an important problem to be addressed.

## Figures and Tables

**Table 1:** Business and technology trends on fundamental education.

Business Trends	Technology Trends
The shift to active learning	Artificial intelligence
Change the definitions of schools and student’s success	Virtual/augmented reality
Personalized learning	Digital assessment
Analytics everywhere	Adaptive learning
Privacy and trust	Digital ecosystems

**Table 2:** Business and technology trends on higher education.

Business Trends	Technology Trends
Competency-based education	Open micro-credentials
Reinventing credentials	Digital assessment
Analytics everywhere	Predictive analysis
Ranking	Adaptive learning
Breaking boundaries	VR/AR comeback
Revenue diversification	Hybrid integration platforms
Increasing political intervention	Institutional video management
Innovative learning spaces	Artificial intelligence
Personalization	Listening and sensing technologies
Student recruiting	Robotic telepresence

**Table 3:** Stakeholders and educational governance drivers.

Stakeholders	Needs	Drivers
Government	Quality education. Well-formed citizen.	Education policies, approval rate, evaluation results, public acceptance.
Society	Well-formed citizen. Economically productive.	Job market, individual and collective desires, economy, technological trends.
School Management	Well-formed citizen. Individual capable of performing professionally. Operational efficiency.	Regulatory policies and laws, market positioning, financial health, operational efficiency, learning, technological trends.
Parents	Well-formed children. Children capable of achieve their goals. Feedback. Synergy with teachers.	Learning, communication, technological trends, child training, economics.
Teachers	Reliable information. Learning process. Synergy with parents. Identify improvement points.	Learning, communication, technological trends, teaching methods.
Students	Learn. Being engaged. Feel himself/herself as part of the process.	Learning, engagement, trends, communication.

**Table 4:** Educational research and practice.

Educational Research and Practice	Computational Intelligence Task
Classify Students’ Profile	Classifiers
Analysis and Visualization of Data	Data science
Providing Feedback for Supporting Instructors	Association-rule
Recommendations for Students	Sequential pattern mining
Predicting Student’s Performance	Neural networks
Student Modeling	Bayesian networks
Detecting Undesirable Student Behaviors	Classifiers
Grouping Students	Classifiers and clustering
Social Network Analysis	Collaborative filtering
Developing Concept Maps	Text mining
Constructing Courseware	Existing learning resources
Planning and Scheduling	Combinatorial optimization

**Table 5:** Defined driver and objective.

Driver	Objective
Good student performance	Improve the student success rate

**Table 6:** Characteristics of the school.

Characteristic	Value
Total of classroom	20
Total of students by classroom	28
Total of laboratory	2
Total of computers in laboratory	20
Total of computers with internet access	20
Total of management departments	16
Extra-curricular activities	Technology and entrepreneurship
Age of infrastructure	12 years

**Table 7:** Dataset and attribute types. These attributes are used to explain images from 7 until 14.

Name	Description	Type	Category	Class
NLP	Portuguese language grade	Continuous	Target	
FLP	Portuguese language absenteeism	Continuous	Target	
NEF	Physical education grade	Continuous	Target	
FEF	Physical education absenteeism	Continuous	Target	
NAR	Arts grade	Continuous	Target	
FAR	Arts absenteeism	Continuous	Target	
NMA	Math grade	Continuous	Target	
FMA	Math absenteeism	Continuous	Target	
NCC	Science grade	Continuous	Target	
FCC	Science absenteeism	Continuous	Target	
NHI	History grade	Continuous	Target	
FHI	History absenteeism	Continuous	Target	
NGE	Geography grade	Continuous	Target	
FGE	Geography absenteeism	Continuous	Target	
NER	Religion grade	Continuous	Target	
FER	Religion absenteeism	Continuous	Target	
NES	Spanish language grade	Continuous	Target	
FES	Spanish language absenteeism	Continuous	Target	
NEN	English language grade	Continuous	Target	
FEN	English language absenteeism	Continuous	Target	
NTE	Technology grade	Continuous	Target	
FTE	Technology absenteeism	Continuous	Target	
FTT	Total of absenteeism	Continuous	Target	
FPE	Percent of absenteeism	Categorical	Target	
Status	Final situation	Categorical	Target	Approved, reproved
MATRICULA	Internal identification	Continuous	Qualifier	
TURMA	Internal division	Categorical	Qualifier	A,B,C
SERIE	Division by content	Categorical	Qualifier	
SEXO	Genre	Categorical	Qualifier	
DATA_NASCIMENTO	Student’s date of birth	Categorical	Qualifier	
IDADE_ALUNO	Student’s age	Continuous	Qualifier	
BAIRRO	Neighborhood	Categorical	Qualifier	
CIDADE	City	Categorical	Qualifier	
ESTADO	State	Categorical	Qualifier	
PAIS	Country	Categorical	Qualifier	
CEP	Zip code	Categorical	Qualifier	
DN	Father’s date of birth	Categorical	Qualifier	
IDADE_PAI	Father’s age	Continuous	Qualifier	
EDUCACAO_PAI	Father’s education	Categorical	Qualifier	
PROFISSAO_PAI	Father’s profession	Categorical	Qualifier	
DN1	Mother’s date of birth	Categorical	Qualifier	
IDADE_MAE	Mother’s age	Continuous	Qualifier	
EDUCACAO_MAE	Mother’s education	Categorical	Qualifier	
PROFISSAO_MAE	Mother’s profession	Categorical	Qualifier	
RENDA_MEDIA_FAMILIA	Family’s income	Continuous	Qualifier	
CATEGORIA_ALUNO	Internal division	Categorical	Qualifier	
TURNO	Class period	Categorical	Qualifier	Matutino, Vespertino
